# Occult Serologically Confirmed Cases of SARS-CoV-2 Coronavirus among the General Population in the Era of the Fourth Vaccination

**DOI:** 10.3390/jcm13164953

**Published:** 2024-08-22

**Authors:** Mori Hay Levy, Neta Cohen, Rotem Marom, Hanoch Goldshmidt, David Zeltser, Michal Mizrahi, Yanay Simhon, Ronni Gamzu, Nadir Arber, Shahar Lev-Ari, Tali Capua, Esther Saiag

**Affiliations:** 1 Department of Epidemiology and Preventive Medicine, Faculty of Medicine, Tel Aviv University, Tel Aviv 6997801, Israel; morilevy87@gmail.com (M.H.L.); leva@tauex.tau.ac.il (S.L.-A.); 2 Faculty of Medicine, Tel Aviv University, Tel Aviv 6997801, Israel; netaco@tlvmc.gov.il (N.C.); rotemma@tlvmc.gov.il (R.M.); hanochg@tlvmc.gov.il (H.G.); davidz@tlvmc.gov.il (D.Z.); michalmi@tlvmc.gov.il (M.M.); yanaysi@tlvmc.gov.il (Y.S.); ronni@tlvmc.gov.il (R.G.); nadira@tlvmc.gov.il (N.A.); esthers@tlvmc.gov.il (E.S.); 3 Pediatric Emergency Department, Dana Dwek Children’s Hospital, Tel Aviv Sourasky Medical Center, Tel Aviv 6423906, Israel; 4 Division of Clinical Laboratories, Tel Aviv Sourasky Medical Center, Tel Aviv 6423901, Israel; 5 Department of Emergency Medicine, Tel Aviv Sourasky Medical Center, Tel Aviv 6423901, Israel; 6 Internal Medicine Department, Tel Aviv Sourasky Medical Center, Tel Aviv 6423901, Israel; 7 Management, Tel Aviv Sourasky Medical Center, Tel Aviv 6423901, Israel; 8 Health Promotion Center and Integrated Cancer Prevention Center, Tel Aviv Sourasky Medical Center, Tel Aviv 6423906, Israel; 9 Department of Information Systems and Operation, Tel Aviv Sourasky Medical Center, Tel Aviv 6423901, Israel

**Keywords:** SARS-CoV-2 infection, asymptomatic disease, occult serologically confirmed cases, anti-N IgG, SARS-CoV-2 health policy, public health, vaccination era

## Abstract

**Background:** Asymptomatic SARS-CoV-2 infection can significantly increase the spread of the COVID-19 pandemic. We aimed to investigate the epidemiological and clinical predictors of occult serologically confirmed SARS-CoV-2 cases among the general population during the fourth vaccination era in Israel. **Methods:** We conducted a cross-sectional study among individuals aged ≥18 years who had not been tested for COVID-19 in the preceding 5 months. Occult serologically confirmed cases were based on the presence of anti-N IgG antibodies. Potential risk factors were examined. Multivariable regression analysis identified independent predictors of subclinical SARS-CoV-2 infection. **Results:** This study included 504 participants. The prevalence of occult serologically confirmed SARS-CoV-2 was 12.5%. Chronic disease was found to be an independent predictor for the absence of occult disease (aOR) 0.4 [95% (CI): 0.18–0.87], *p*-value = 0.02). No significant differences were observed in age, sex, marital status, number of children, vaccination status, or exposure to COVID-19 infection between participants with and without SARS-CoV-2 sub-infection. **Conclusions:** We found a lower prevalence of occult serologically confirmed SARS-CoV-2 cases, compared to previous reports, and a negative correlation between chronic disease and occult SARS-CoV-2. Continued research, surveillance, and intervention strategies are needed to optimize long-term health outcomes and provide valuable insights for public health policymakers and clinicians.

## 1. Background

The COVID-19 pandemic, caused by the novel coronavirus SARS-CoV-2, emerged in late 2019 and rapidly became a global crisis [1]. The virus was first identified in Wuhan, China, and its spread quickly escalated into a worldwide pandemic, leading to unprecedented disruptions across nearly every facet of society [2]. SARS-CoV-2 is the seventh coronavirus strain known to infect humans since the first human coronavirus was identified in the 1960s [3]. By early 2020, the World Health Organization (WHO) declared the outbreak a Public Health Emergency of International Concern and subsequently a pandemic, as the virus had spread globally, affecting millions [4]. The pandemic triggered widespread lockdowns, social distancing measures, and travel restrictions aimed at curbing the spread of the virus [5]. These public health interventions had far-reaching effects on daily life, including disruptions to economies, education systems, and healthcare services. Many geopolitical factors were shown to affect the COVID-19 infection rate such as population density, urbanization, climate and religious diversity of the region. Furthermore, GDP per capita and income inequality had a significant effect [6], the evolving strains of SARS-CoV-2, such as Alpha, Delta, and Omicron, significantly impacted the COVID-19 pandemic by increasing transmissibility, reducing vaccine efficacy, and necessitating frequent updates to public health measures and vaccines [7]. These variants also led to shifts in disease severity and highlighted the need for ongoing genomic surveillance and adaptive response strategies [7].

SARS-CoV-2 infection can present with a wide range of clinical manifestations, from subclinical infection to severe, multisystem organ failure and death [8,9,10]. Asymptomatic SARS-CoV-2 infection plays an important role in driving the spread of the pandemic [11]. This is because individuals who are infected but show no symptoms can unknowingly transmit the virus to others, making it challenging to identify and isolate cases promptly. This silent transmission contributes to rapid and widespread outbreaks, complicating efforts to control the pandemic and underscoring the importance of widespread testing and preventive measures. Knowledge of the total number of positive cases is key to the formulation of effective policies which can lead to the prevention of spread as well as to damage control [8]. Estimating the number of positive cases in the general population is crucial for strategic decision-making processes [12]. Asymptomatic infection may present additional challenges to managing the overall disease burden in individuals with comorbidities due to the possibility of long COVID [13]. Furthermore, asymptomatic HCWs pose a significant threat to high-risk persons around them. PPE donning and doffing protocols have high potential for failure and as such, asymptomatic HCWs can infect immunocompromised persons [14]. Despite extensive testing and surveillance efforts, a substantial portion of SARS-CoV-2 infections remain undiagnosed or unreported, particularly those that are asymptomatic or mildly symptomatic [12].

In January 2022, the Israeli Ministry of Health reported an 11.5% rate of positive RT-PCR test results for asymptomatic travelers arriving in Israel from abroad [15]. Several studies explored the rates of SARS-CoV-2 subclinical infection [15,16], and reported positive test rates of 0.8–1.7% in the general population and that 45% of them were asymptomatic [17]. Moreover, 87% of pregnant women who were screened before labor and found to be positive for COVID-19 were asymptomatic [18]. Earlier studies explored mainly occult SARS-CoV-2 rates but, to the best of our knowledge, no study sought to determine risk factors for occult serologically confirmed SARS-CoV-2 cases. Understanding the burden of undiagnosed occult SARS-CoV-2 cases among the general population is essential for accurately assessing population immunity, predicting epidemic trajectories, and evaluating the effectiveness of public health measures such as social distancing, mask mandates, and vaccination campaigns. We therefore aimed to explore the epidemiological and clinical predictors for occult serologically confirmed SARS-CoV-2 cases among the general population in the era of the fourth vaccination in Israel and suggest how understanding these predictors can help with future pandemic waves.

## 2. Methods

### 2.1. Study Design and Population

The study population consisted of 504 individuals aged 18 years or older who had not been diagnosed with COVID-19 in the five months preceding study entry (1 March–30 June 2022). All participants successfully completed the required questionnaire, as failure to do so was an exclusion criterion. Additionally, individuals with an active or recent COVID-19 diagnosis within the previous five months were excluded from participation. Recruitment was conducted through a variety of media channels, including online platforms, as well as targeted invitations extended to individuals visiting the Health Promotion Center at Tel Aviv Medical Center. No incentives were provided to participants nor were any other actions taken to enhance access to participation.

### 2.2. Assessment of Occult Serologically Confirmed Cases

Occult serologically confirmed cases were defined as the presence of anti-N IgG antibodies in blood samples of study participants who had not previously been diagnosed with COVID-19 within the 5 months prior to blood samples being taken, indicating past exposure to SARS-CoV-2 that was asymptomatic or undetected. Study participants were requested to complete a questionnaire. The questionnaire included behavioral variables that were reportedly associated with SARS-CoV-2 coronavirus infection such as travel history and contact with infected individuals [19,20,21], as well as demographic characteristics.

### 2.3. Measurements

Anti-N IgG levels were determined with Abbott’s SARS-CoV-2 IgG reagent kit 06R90. The assay is a chemiluminescent micro particle immunoassay performed on the Anility system (Abbott, Chicago, IL, USA). Antibody levels are provided as an index sample/calibrator with a threshold value of 1.4 for a positive result. The choice of Abbott’s SARS-CoV-2 IgG reagent kit was based on its validated performance and high sensitivity for detecting anti-N IgG antibodies.

### 2.4. Outcome

The primary outcome was defined as a positive anti-N-IgG test result in a participant who had not been diagnosed with COVID-19 in the previous 5 months, which was used to identify individuals with undetected prior infection.

### 2.5. Potential Predictors of Severe Outcome

Based upon the findings of a literature review [9,22,23,24,25,26,27] and the clinical experience of the investigators, we investigated potential demographic and clinical risk factors for occult serologically confirmed SARS-CoV-2 cases.

### 2.6. Data Analysis

Data entry and analysis were performed with SPSS Statistics, version 28 (SPSS Inc., Chicago, IL, USA), ensuring accuracy and reliability in statistical computations. A two-tailed *p*-value < 0.05 was considered statistically significant. All parameters with a significance level of *p* < 0.2 and those considered clinically relevant in the univariate analysis were inserted into the multivariable model. Odds ratios (ORs) with 95% confidence intervals (95% CIs) were determined to quantify the strength of the associations in the multivariate regression model.

## 3. Results

### 3.1. Study Cohort Characteristics

Demographics: A total of 504 adults participated in the study, with a mean age of 48.5 ± 12.5 years. The cohort comprised 241 females (47.8%). The majority of participants (*n* = 337, 66.9%) were married. On average, participants had 1.9 ± 1.3 children (see Table 1).

Vaccination Status: A total of 349 participants (69.2%) had received three doses of the BNT162b2 vaccine (see Table 1).

Exposure History: Two hundred thirty-four participants (46.5%) reported exposure to a known COVID-19 patient in the preceding 5 months but were not diagnosed with COVID-19 themselves (see Table 1).

### 3.2. Serological Findings

Occult SARS-CoV-2 Cases: Out of the 504 participants, 65 (12.5%) tested positive for anti-N IgG antibodies, indicating occult serologically confirmed SARS-CoV-2 cases.

### 3.3. Comparative Analysis

We compared the group of patients who were serologically confirmed with SARS-CoV-2 and the group of patients who were not found to have SARS-CoV-2 sub-infection. Sixty-five (12.5%) patients had positive anti-N IgG antibody test results and were defined as having occult serologically confirmed SARS-CoV-2 cases.

Demographic Comparisons: There were no significant differences between participants with occult SARS-CoV-2 cases and those without in terms of age, sex, marital status, or number of children (*p*-value > 0.05) (see Table 2). The number of doses of the BNT162b2 vaccine was also not significantly different between the two groups. Importantly, there were significantly higher rates of participants with chronic disease who did not have confirmed SARS-CoV-2 sub-infection compared to the group of participants with occult serologically confirmed cases (29.9% vs. 15.9%, respectively, *p*-value = 0.02). The rates of specific comorbidities between the two groups, however, were comparable (Table 2).

### 3.4. Multivariable Analysis

Predictors of occult SARS-CoV-2: In the multivariable model, the presence of chronic disease was identified as an independent predictor for the absence of occult SARS-CoV-2 cases. The adjusted odds ratio (OR) for the absence of occult SARS-CoV-2 cases in individuals with chronic disease was 0.4 (95% CI: 0.18–0.87, *p*-value = 0.02) (see Table 3).

## 4. Discussion

Our cross-sectional study found a 12.9% prevalence of occult serologically confirmed SARS-CoV-2 cases in the general population during the era of the fourth COVID-19 vaccination. This rate is considerably lower than the rate of 20% reported by the Israel Ministry of Health in a survey of pre-flight tests of asymptomatic travelers [16]. This difference, however, can hypothetically stem from the possibility that some travelers were actually symptomatic but failed to report symptoms and were discovered as being infected by the virus by those pre-flight tests, while our study participants were predominantly asymptomatic or tested negative on PCR tests. Moreover, the high rate of vaccination coverage in our study population may have contributed to the lower prevalence.

We found the presence of a chronic disease to be an independent protective factor against occult serologically confirmed SARS-CoV-2 cases. This finding may be explained by the known effect of comorbidity in active SARS-CoV-2 disease [21]. Moreover, fatal cases of COVID-19 are more prevalent among patients with comorbidities [28]. Comorbidities are known to increase the likelihood of severe COVID-19 disease, resulting in a lower prevalence of asymptomatic cases within the general population [29,30,31,32]. Moreover, it has been observed that individuals with chronic conditions exhibit a higher inclination to comply with medical recommendations and engage in health-seeking behavior [33,34]. This indicates that the presence of underlying health conditions may contribute to a greater adherence to prescribed medical interventions and preventive measures during the COVID-19 pandemic.

Sex was not associated with occult serologically confirmed SARS-CoV-2 cases in our study, but we found conflicting results in the literature [10,30,31]. Similar to our current results, a study of 2590 healthcare workers found 31.6% IgG-positive, with no differences in age, sex, or previous diseases to those who were IgG-negative [31].

Methodologically, this study contributes to the field of clinical epidemiology by introducing innovative approaches for identifying and quantifying occult serologically confirmed cases. The implications of this study extend beyond the individual level to public health policies. The identification of subclinical infection and the potential impact of the fourth vaccination call for a reassessment of strategies aimed at mitigating the long-term health effects of SARS-CoV-2.

There are several limitations of our study that bear mention. The findings are specific to a population in Israel during the specified time period, thereby limiting generalizability. The results may not be applicable to other countries or populations with different demographic characteristics, vaccination rates, or healthcare systems. There is also the possibility of a selection bias stemming from the study participants having been recruited through media publications and invitations when they underwent routine health checks. The use of self-reported information about comorbidities, symptoms, and exposure to COVID-19 introduces the potential for recall bias or misinformation, which could impact the accuracy of the results.

## 5. Conclusions

In conclusion, our clinical study has shed light on the prevalence and predictors of occult serologically confirmed SARS-CoV-2 cases among the general population during the era of the fourth COVID-19 vaccination in Israel. The following key conclusions and their implications for healthcare policies can be drawn from our findings:

Prevalence of Occult SARS-CoV-2 Infection: This study revealed a 12.9% prevalence of occult serologically confirmed SARS-CoV-2 cases, a rate lower than reported in certain subgroups such as asymptomatic travelers. The lower prevalence in our study may be attributed to factors such as vaccination coverage and the predominance of asymptomatic or PCR-negative individuals in our cohort.

Chronic Disease: Notably, the presence of chronic disease emerged as an independent protective factor against occult serologically confirmed SARS-CoV-2 cases. This finding suggests that individuals with chronic conditions may exhibit a lower prevalence of asymptomatic cases, potentially due to their heightened adherence to medical recommendations and preventive measures. This could influence clinical monitoring and preventive strategies for these populations.

Vaccination Impact: The study underscored the high vaccination coverage (69.2% received three doses) in the study population and its potential contribution to the lower prevalence of occult serologically confirmed SARS-CoV-2 cases. Understanding the dynamics of vaccination impact on pathogen growth and virulence selection is crucial for shaping effective public health strategies.

Sex and Occult Morbidity: Unlike some conflicting literature, our study did not find an association between sex and occult serologically confirmed SARS-CoV-2 cases. This highlights the complexity of demographic factors in SARS-CoV-2 infection and the need for further research to elucidate these relationships.

Public Health Implications: Methodologically, our study introduces innovative approaches for identifying and quantifying occult serologically confirmed cases, contributing to the field of clinical epidemiology. The implications extend beyond the individual level to public health policies, emphasizing the importance of reassessing strategies to mitigate the long-term health effects of SARS-CoV-2.

Limitations and Generalizability: Acknowledging the study’s limitations, including its specificity to the Israeli population and the specified time period, calls for cautious generalizability. Despite these limitations, the study provides valuable insights into the dynamics of occult serologically confirmed SARS-CoV-2 cases.

Healthcare Policy Recommendations:

The findings from our study have direct implications for shaping healthcare policies:

Targeted Interventions for High-Risk Groups: Policymakers can use the information about chronic diseases to design targeted interventions for high-risk groups, emphasizing the importance of preventive measures and vaccination.

Optimizing Vaccination Strategies: Understanding the impact of vaccination coverage on occult infections informs vaccination strategies. Policymakers may consider optimizing vaccination campaigns to enhance coverage and mitigate the spread of SARS-CoV-2.

Enhanced Surveillance and Research: The study highlights the need for continued surveillance and research efforts to monitor the prevalence of occult infections, identify evolving risk factors, and tailor public health responses accordingly.

By recognizing and addressing occult serologically confirmed SARS-CoV-2 cases, policymakers can optimize long-term health outcomes, enhance public health preparedness, and contribute to the global effort to combat the ongoing pandemic.

## Figures and Tables

**Table 1:** Characteristics of the study participants.

Characteristic	*n* = 504
Mean age ± Standard Deviation (years)	48.5 ± 12.5
Female	241 (47.8%)
**Family status**	
Single	118 (23.4%)
Married	337 (66.9%)
Divorced	41 (8.1%)
Widowed	8 (1.6%)
Children, mean ± SD	1.9 ± 1.3
Exposure to SARS-CoV-2	234 (46.4%)
**Chronic medical conditions**	142 (28.1%)
**Vaccination status**	
Not vaccinated	6 (1.2%)
1 dose of BNT162b2	4 (0.8%)
2 doses of BNT162b2	42 (8.3%)
3 doses of BNT162b2	349 (69.25%)
4 doses of BNT162b2	103 (20.4%)
Previously tested negative	234 (46.4%)
Suspected COVID-19 symptoms *	177 (35.1%)

* Unconfirmed self-reported.

**Table 2:** A comparison of epidemiological and clinical characteristics of patients according to occult COVID-19 morbidity.

Characteristic	No Occult Morbidity (*n* = 441)	Occult Morbidity (*n* = 63)	*p*-Value
Sex (female)	210 (47.6)	29 (46.0)	0.89
Age	48.8 ± 12.5	47.3 ± 11.7	0.18
Marital status, married	302 (68.5)	37 (58.7)	0.11
Children, *n*	1.9 ± 1.3	1.8 ± 1.4	0.35
Vaccination status			
Vaccinations, *n*	3.0 ± 0.64	3.0 ± 0.67	0.62
Not vaccinated	1 (1.6)	5 (1.1)	0.55
1 dose of BNT162b2	1 (1.6)	3 (0.7)	0.41
2 doses of BNT162b2	4 (6.3)	38 (8.6)	0.80
3 doses of BNT162b2	46 (73.0)	303 (68.7)	0.56
4 doses of BNT162b2	92 (20.9)	11 (17.5)	0.61
Chronic medical conditions	132 (29.9)	10 (15.9)	0.02
Immunocompromised	7 (1.6)	0 (0.0)	0.60
Asthma	5 (1.1)	0 (0.0)	>0.99
DM	11 (2.5)	2 (3.2)	0.69
Hypothyroidism	15 (3.4)	2 (3.2)	0.64
Hypercholesterolemia	43 (9.8)	3 (4.8)	0.24
HTN	27 (6.1)	1 (1.6)	0.23
Positive viral symptoms in the preceding 5 months	151 (34.2)	26 (41.3)	0.32
Known exposure to a COVID-19-infected person	199 (45.1)	35 (55.6)	0.13

Values are reported as mean ± SD unless indicated otherwise. The numbers in the parentheses are percentages. DM—diabetes mellitus; HTN—hypertension.

**Table 3:** A multi-regression model to predict occult morbidity.

	aOR	95% CI	*p*-Value
Marital status, married	1.05	0.8–1.3	0.71
Sex, female	0.82	0.4–1.4	0.49
Age, years	1.0	0.9–1.0	0.67
**Comorbidity**	**0.4**	**0.1–0.8**	**0.02**
A confirmed exposure to COVID-19 patients in the last 5 months	1.4	0.8–2.5	0.17
Viral symptoms during the last 5 months	1.2	0.7–2.2	0.43
Number of vaccinations	0.99	0.6–1.5	0.98

## Data Availability

Name of the registry: MOH. Trial registration number: MOH_2024-01-10_013177. Date of registration: retrospectively registered—10 January 2024. URL of trial registry record: https://my.health.gov.il/CliniTrials (accessed on 18 July 2024). The datasets used and/or analyzed during the current study are available from the corresponding author upon reasonable request.
